# Decolonising qualitative research with respectful, reciprocal, and responsible research practice: a narrative review of the application of Yarning method in qualitative Aboriginal and Torres Strait Islander health research

**DOI:** 10.1186/s12939-022-01738-w

**Published:** 2022-09-13

**Authors:** Michelle Kennedy, Raglan Maddox, Kade Booth, Sian Maidment, Catherine Chamberlain, Dawn Bessarab

**Affiliations:** 1 College of Health Medicine and Wellbeing, The University of Newcastle, Callaghan, NSW Australia; 2 Hunter Medical Research Institute, New Lambton Heights, NSW Australia; 3 National Centre for Epidemiology and Public Health, The Australian National University, Canberra ACT, Australia; 4 School of Population and Global Health, Centre for Health Equity, University of Melbourne, Melbourne, VIC Australia; 5 Judith Lumley Centre, School of Nursing and Midwifery, La Trobe University, Melbourne, VIC Australia; 6 Ngangk Yira Research Centre for Aboriginal Health and Social Equity, Murdoch University, Perth, WA Australia; 7 Centre for Aboriginal Medical and Dental Health, UWA Medical School, Crawley, WA Australia

**Keywords:** Qualitative, Aboriginal health, Indigenous methods, Critical review, Yarning method

## Abstract

**Background:**

Indigenous academics have advocated for the use and validity of Indigenous methodologies and methods to centre Indigenous ways of knowing, being and doing in research. Yarning is the most reported Indigenous method used in Aboriginal and Torres Strait Islander qualitative health research. Despite this, there has been no critical analysis of how Yarning methods are applied to research conduct and particularly how they privilege Indigenous peoples.

**Objective:**

To investigate how researchers are applying Yarning method to health research and examine the role of Aboriginal and Torres Strait Islander researchers in the Yarning process as reported in health publications.

**Design:**

Narrative review of qualitative studies.

**Data sources:**

*Lowitja Institute* LitSearch January 2008 to December 2021 to access all literature reporting on Aboriginal and Torres Strait Islander health research in the *PubMed* database. A subset of extracted data was used for this review to focus on qualitative publications that reported using Yarning methods.

**Methods:**

Thematic analysis was conducted using hybrid of inductive and deductive coding. Initial analysis involved independent coding by two authors, with checking by a third member. Once codes were developed and agreed, the remaining publications were coded and checked by a third team member.

**Results:**

Forty-six publications were included for review. Yarning was considered a culturally safe data collection process that privileges Indigenous knowledge systems. Details of the Yarning processes and team positioning were vague. Some publications offered a more comprehensive description of the research team, positioning and demonstrated reflexive practice. Training and experience in both qualitative and Indigenous methods were often not reported. Only 11 publications reported being Aboriginal and/or Torres Strait Islander led. Half the publications reported Aboriginal and Torres Strait Islander involvement in data collection, and 24 reported involvement in analysis. Details regarding the role and involvement of study reference or advisory groups were limited.

**Conclusion:**

Aboriginal and Torres Strait Islander people should be at the forefront of Indigenous research. While Yarning method has been identified as a legitimate research method to decolonising research practice, it must be followed and reported accurately. Researcher reflexivity and positioning, and Aboriginal and Torres Strait Islander ownership, stewardship and custodianship of data collected were significantly under detailed in the publications included in our review. Journals and other establishments should review their processes to ensure necessary details are reported in publications and engage Indigenous Editors and peer reviewers to uphold respectful, reciprocal, responsible and ethical research practice.

**Supplementary Information:**

The online version contains supplementary material available at 10.1186/s12939-022-01738-w.

## Introduction

Indigenous peoples have undertaken research since time immemorial, as evidenced in our continued survival prior to, and post colonisation and contemporary coloniality [1]. However, Indigenous peoples have long been researched by non-Indigenous peoples as mere objects, without prior consent to the research and commonly without meaningful engagement, or access to the results. In colonised countries, research has been utilised as a tool to dehumanise Indigenous peoples [1]. In Australia, research was used as a tool to justify Terra Nullius, “no man’s land”, the grounds on which the continent was taken by the crown with no negotiation or treaty offered [2] to the 500 Aboriginal and Torres Strait Islander nations [2, 3] that have lived on this land, now called Australia, for over 60,000 years [4]. Research such as that conducted by D. J Cunningham (1889) “The Spinal Curvature in an Aboriginal Australia” which reported *“…In these particulars the Australian spine resembles somewhat the spine of a Chimpanzee”* [5] was used to de-humanise Aboriginal people, disrupting culture and cultural practice [6]. Findings were applied to understand the antithetical other and to justify the claim of Australia as uninhabited lands. Further, Darwin used such data to support his theory of evolution, arguing that the “natives” (sic) were the living example of the difference in degree between humans and apes [7]. Australia was colonised on a racially imperialistic basis which has been embedded through coloniality [8]. In the words of Linda Tuhiwai Smith:

*“This collective memory of imperialism has been perpetuated through the ways in which knowledge about indigenous peoples was collected, classified and then represented in various ways back to the West, and then through the eyes of the West, back to those who have been colonized”.* [1] (p. 30)

In response to the colonial legacy of research and its dirty [1] reputation among Indigenous people, the use of Indigenous research methodologies and methods to aid in decolonising the research process have been advocated for, and by Indigenous academics domestically and internationally [1, 9]. Decolonising approaches recognise that the way of knowing has been historically and institutionally contrived in a Western construct, [10] and that Indigenous methodologies and methods can be used to shift the research paradigm and privilege Indigenous ways of knowing, being and doing. Indigenous ways of knowing, being and doing are shaped by our relationality. Relationality to each other, our lands, our knowledge systems and our storylines [11].

Morton Robinson describes;

*“Relationality is an inextricable part of our sovereign knowledges, informing our scholarship to produce innovative social research. As a presupposition it shapes ways of knowing, being and doing to be connected is to know, and knowing is embodied in social relations and bloodline to country, determined by ancestors and creator beings that guide who can be a knower and of what knowledges”*. [11]

Relationality shapes Indigenous methodologies, informing the ways in which research is conceptualised, designed, conducted, analysed and disseminated. As such the ways in which Indigenous methodologies are applied will vary depending on the relationality, social and cultural positioning of the researcher and peoples involved. In an example outlining Indigenist Research Methodology, Aboriginal scholar Rigney states:

*“Indigenist research is research by Indigenous Australians whose primary informants are Indigenous Australians and whose goals are to serve and inform the Indigenous struggle for self-determination”.* [12] (p. 118)

Research methods are then applied by the researcher to undertake the research, the ‘doing’. An international systematic review by Drawson et al. reported three key components to Indigenous Research Methods:

1. Researchers must situate themselves and the Indigenous Peoples with whom they are collaborating in the research process
2. The inclusion of Indigenous Peoples in the research process in a way that is respectful, reciprocal, and decolonizing and preserving of self-determinism, and
3. Prioritization of Indigenous ways of knowing [13]

These key components of Indigenous research methods coincide with the established standards for conducting ethical research with Aboriginal and Torres Strait Islander people, such as the National Health and Medical Research Council (NHMRC) Values and Ethics Guideline, [14] the Australian Institute of Aboriginal and Torres Strait Islander Studies (AIATSIS) Code of Ethics [15] and the Aboriginal Health and Medical Research Council (AHMRC) Key Principles [16].

**Fig. 1:**
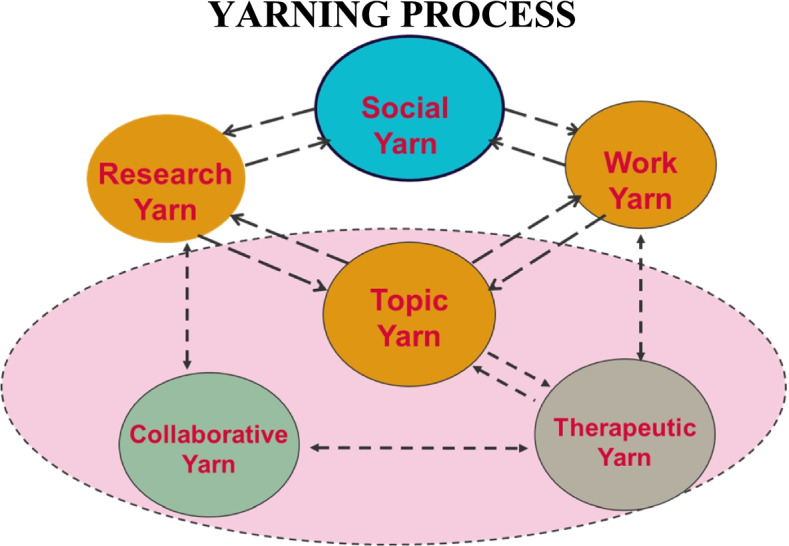
A working depiction of the Yarning Process as developed by Dawn Bessarab

Yarning in Indigenous qualitative research is one method being used in Australia and internationally, [17] and has been recommended for use in Aboriginal and Torres Strait Islander health research [9] to privilege Indigenous ontologies [18]. Yarning has been used in recent research as a way to safely engage with participants to explore research questions relating to the topic of the study. The cultural safety of yarning enables sensitive issues to emerge as it fosters agency among participant(s) including the ability to disclose information at their own discretion [14]. Yarning is led by the researcher where the participant is encouraged to tell their story from the position of their lived experience. Whilst the research topic yarn does not follow a pre-determined set of questions, it does include a yarning topic guide relating to the research that the researcher is listening for in the story. Yarning does not follow the formal conventions of research interviewing and can weave in and out of the yarning story where the role of the researcher is to listen for cues related to the research topic. Yarning as a research method must also draw on cultural protocols and practices that are relevant to the people’s involved. Yarning draws on relationality through processes of the Social, Work and Research Topic Yarn which can inform either Collaborative Yarning or Therapeutic Yarning as presented in Fig. 1 [17]. Relationality of the Yarn is paramount to producing rich data [19]. It is reasonable to expect that Indigenous Standpoint generates deeper relationality, through shared experience and understandings of the Yarn [19].

Whilst there appears to be consensus among Indigenous and non-Indigenous scholars on the need for decolonising research approaches and the importance of utilising Indigenous research methodologies and methods, we could not find literature specifically relating to how these are pragmatically applied to the conduct of research. The NHMRC directs researchers to conduct ethical research in line with six core values: Spirit and integrity, Cultural continuity, Equity, Respect, Reciprocity, and Responsibility [14]. Our Indigenous-led team sought to investigate how researchers are applying Yarning method to ethical health research. We then examined and analysed the Yarning process, including the role of Aboriginal and Torres Strait Islander people as reported in health publications.

## Research team

Our lived experiences and coming to understand our relation to the world is complex, dynamic, but fundamentally important, as we recognise that Indigenous peoples ways of knowing, being and doing are relational [11]. This includes, but is limited to what is known, how it is known; the nature and embodiment of our realities, encapsulating what exists, what is possible, [20] and how we relate to our respective programs of research.

The majority Indigenous research team is led by a Wiradjuri woman MK with disciplinary training in social science/social work and Indigenous knowledges connected to Wiradjuri, Worimi and Awabakal country. KB is a non-Indigenous researcher, experienced in qualitative health research with a social science background. SM is a Noongar woman with disciplinary training in exercise and sport science and a current medical student. CC is a Palawa woman of the Trawlwoolway clan with training in midwifery, nursing and public health, and experienced in mixed methods research. DB is an Indigenous researcher from the Bard and Yjindabandi nations in Western Australia and is a senior social worker with extensive background and expertise in Aboriginal health research and methodologies specifically yarning. RM is from the Bagumani (Modewa) Clan in Papua New Guinea, with training and experience in public health and epidemiology.

Our research team embodies over 200 years of lived Indigenous experience and over 60 years’ experience conducting qualitative research in Aboriginal and Torres Strait Islander health.

## Methodology

This review forms part of a larger project exploring the conduct of Aboriginal and Torres Strait Islander health research, led by an Aboriginal and Torres Strait Islander research team. A primary scoping review was conducted of all Aboriginal and Torres Strait Islander health research published since the establishment of the Closing the Gap campaign [21]. This parent review included 2,150 articles and is published elsewhere [22]. When conducting this review, the authors sought to extract information on the reported use of Indigenous research methodologies and found 5% of articles reported using Indigenous methodologies and/or methods. Indigenous methodologies/methods were predominately reported in qualitative papers. This was not published in the parent review.

This review of Yarning aims to answer two research questions:

1. How are researchers applying the Yarning method in qualitative health research?
2. What is the role of Aboriginal and Torres Strait Islander researchers in the Yarning process, as reported in health publications?

### Design and inclusion criteria

The parent review applied a systematic literature search via the Lowitja Institute website using the search tool *Lowitja.search* to access all Aboriginal and Torres Strait Islander health literature in the *PubMed* database. The selected topics in the database were “all” and “Aboriginal and Torres Strait Islander”. Publications were included if they presented original data on Aboriginal and Torres Strait Islander health in Australia and were published between January 2008 and December 2020. The search was updated to include publications until December 2021. Publications that were identified as using qualitative methods for data collection and analysis were assessed. From this, those that reported using Yarning method were included for analysis in this review.

### Level of reporting assessment

We assessed the level of reporting in the selected publications using a purpose-built tool to examine Aboriginal and Torres Strait Islander engagement and oversight of the research. The tool was developed as informed by our research questions, ethical research guidelines and an established Aboriginal and Torres Strait Islander quality appraisal tool [23]. The tool was developed due to the timeframe of included publications, and an acknowledgment of the lack of reporting guidelines for ethical research practice with Indigenous peoples prior to 2019 [24]. The tool has six categories; (1) Aboriginal and Torres Strait Islander engagement in development of the research, (2) Aboriginal and Torres Strait Islander engagement in data collection, (3) experience of researchers reported, (4) Aboriginal and Torres Strait Islander engagement in the analysis, (5) Aboriginal and Torres Strait Islander specific ethics approval granted, and (6) whether the original method publication was cited. Two authors (MK, KB) independently reviewed each document and ranked each publications level of reporting high (5–6), medium (3–4) or low (0–2). This assessment was not used to exclude studies or inform analysis, but rather it was used for Collaborative Yarning among the authorship team which is reflected in the discussion.

### Data analysis

Full text publications were imported into NVivo software for analysis. Three members of the research team who conducted the quality appraisal (KB, MK & SM) engaged in Collaborative Yarning with all authors (MK, RM, KB, SM, CC & DB) to inform the analysis.

Thematic analysis, as outlined by Braun & Clarke, [25] was used to examine how researchers apply Yarning method in qualitative health research (MK & KB). The initial phase included familiarisation with the data. The team members involved in data coding (KB, SM & MK) have been immersed in the data throughout the primary review, and by reading and sorting each qualitative publication that reported using Yarning methods during the inclusion phase. The senior author DB familiarised the data by reading a sample of publications identified by MK. The sample of papers were selected, including a variety of reporting levels, to inform Collaborative Yarning practice to unpack the different perceptions in reporting of Yarning methods. Further, this approach assisted to ensure consistency between the authorship team, actively facilitating discussion on different points of view. The team members met and engaged in Collaborative Yarning to discuss the data after reading the selected publications on how Yarning was reported, and how it was being analysed according to the research questions.

Similar to Fereday, [26] thematic analysis was approached through a hybrid of inductive and deductive coding. As noted in our first research question, the role of Aboriginal and Torres Strait Islander people was a key component to our analysis. Codes were developed deductively from our research questions as we sought to draw out the role of Aboriginal and Torres Strait Islander people in the Yarning process. These codes included: “Aboriginal Involvement”, “Analysis approach”, “Framework Methodology”, and “Yarning Processes”. While these were not necessarily “pre-conceived” by the coders (MK & KB), they were broadly discussed prior to coding in relation to the research questions and were then sought out by the researchers. In conjunction with the initial deductive codes that were drawn out to address parts of the research aims, the coding process was predominantly inductive. Inductive coding was used to examine the integral components of Yarning processes, justification of method and the way that these methods, processes and involvement were being reported. MK & KB independently coded the same three publications before meeting to discuss initial themes. The authors found that overall, coding was similar, with some variations on wording to describe themes. After agreement, MK & KB continued to code a further five of the same publications before meeting again to compare. Any conflicts were discussed until agreement was reached, although disagreements were limited. SM cross checked codes and contributed to discussions of clarity of definitions. MK & KB coded an additional seven publications for comparison, before KB went on to singularly code the remaining publications. SM then reviewed all publications and codes for consistency and agreement to ensure all paper were coded independently and in duplicate.

## Results

**Fig. 2:**
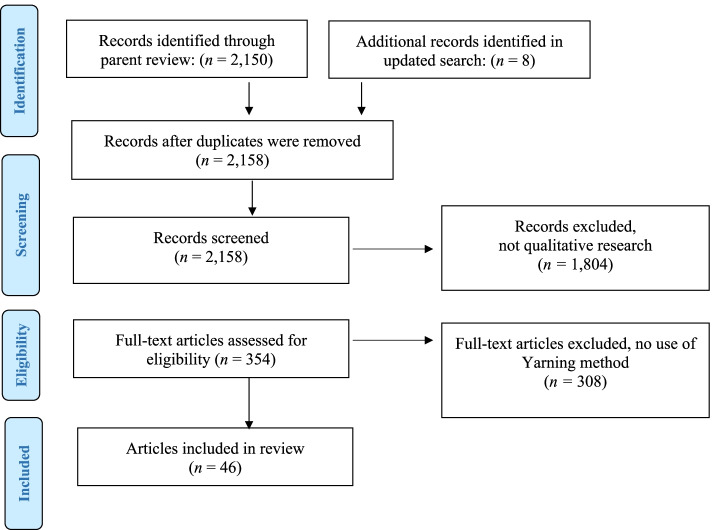
PRISMA Flow diagram of study selection process

Search results are outlined in Fig. 2 using the Preferred Reporting Items for Systematic Reviews and Meta-Analysis (PRISMA) four-phase flow diagram. The total 2,150 papers in the parent reviewed were screened, an updated search was conducted and found 8 new qualitative research papers. *N* = 354 papers reported use of qualitative methods, Yarning method was reported in *n* = 46 papers and were included in analysis.

Of the 46 included publications, 15 were considered high level reporting across focus area, 19 medium level reporting, and 12 low level reporting. We elaborate on the intricacies of this throughout the result section.

We found that researchers are reporting the application of the Yarning method to qualitative research methodology in a wide variety of ways. Both Aboriginal and non-Aboriginal researchers and research teams are using the method, collecting data and involved in analysis.

The following sections will be presented in a manner that addresses the research questions, by detailing how Yarning is being applied to qualitative health research as reported by the included publications, and what role Aboriginal and Torres Strait Islander play in the research process.

### How Yarning is being applied to qualitative health research

Our analysis of the 46 publications showed a variety of reporting on the way that Yarning is applied to Aboriginal and Torres Strait Islander qualitative health research. We begin our critical analysis into this, by first looking at *why* researchers report using Yarning, followed by *how* they report using Yarning and *how* they situate themselves as qualitative researchers and their team in the research process.

#### Why Yarning?

Reasoning for using Yarning method varied across publications. It was predominantly considered to provide a culturally safe and sensitive data collection process [27–38] that privileged Indigenous knowledge systems through connection and relationships [27, 31]. Enabling two-way knowledge sharing; [27, 37, 39] using narratives; [40, 41] storytelling [27, 28, 31, 34, 37, 41–45] and addressing unequal power relationships were most commonly used to justify why Yarning was used, as it is a research process that acknowledges and builds on cultural protocols. One publication stated Yarning was the preferred research method for the older Aboriginal community (see Table 2, 1.3) [46].

Yarning was reported to be an Indigenist research practice [28] that allows for more flexibility than other interviewing approaches [27]. Yarning method was considered to provide an environment that fosters rapport with participants, open discussion, and allow for participant-led research to co-create knowledge and privilege Indigenous voices.

#### How Yarning processes are being reported

Details regarding the specifics of the way that Yarning was conducted were often vague. For example, many publications simply stated that Yarning took place, without detail on the settings or the conversations that took place. However, some publications described various components of yarning, such as Therapeutic or Social yarning. For example, “*In this study, social yarning was used at the beginning of conversations with young people to establish a connection not strongly associated with the actual purpose of the yarn.”* [27] (see Table 2, 2.1).

Most publications reported using audio recording and transcribing to collect data, however, note taking was also reported as a form of data collection [27, 47]. Note taking replaced audio recording to reduce potential harm and was deemed culturally safe in some instances. Mostly, it was due to consent not being obtained by participants who preferred note taking. One publication recognised that starting the recorder had the potential to break the flow of the yarn, so continued taking notes instead.

Eleven publications provided their entire interview guide [31, 34, 39, 45, 48–54]. Others provided a sample of example questions, [40, 44, 55–57] while others briefly described areas explored during Yarning [27, 28, 39, 43, 47, 54, 58–61]. One publication noted that there was *“no set interview guide and that participants were encouraged, with consistent prompts to ‘yarn’ about their experiences with renal service providers”* [62]. Some descriptions were minimal stating guides either had minimal questioning or use of probes [42] or simply stated that they were semi structured.

Some of the more comprehensive papers provided insight into seating arrangement, reimbursement to participants, and provision of meals. One publication described the seating arrangement as a component to providing a safe environment that allowed the researcher to observe body language and non-verbal cues (see Table 2, 2.4) [27]. Eight publications reported reimbursement to participants, which were usually vouchers of between $20 and $50 for their time in participating in the study [40, 49–52, 63–65]. Five publications reported incorporating a meal within the data collection. [32, 38, 45, 63, 65] Study sizes for individual Yarning was reported to include between 4 [49] and 74 participants [28] with minimal justification for the choice of numbers. Yarning circles were often applied across community settings with each Yarning circle including between 5–17 participants at each individual circle. All but one [66] of the publications (*n* = 45) reported the sample size.

Publications reported using varied sampling approaches including *convenient sampling,* [41] and *opportunistic sampling* [42], usually through routine health care [27, 41, 51, 58, 67]. However most papers reported *purposeful sampling*, [27–29, 34, 39, 47, 48, 55, 56, 59–61, 68] including through key community Elders and representatives [33, 50, 69].

Timeframes when conducting Yarning varied significantly from 10-30 min [27] up to 2.5 h, [44, 55, 56] more generally publications reported 40-60 min. Yarning circles were often reported as generally held at a time and place suitable to the participant [58] with use of community outdoor settings or participants houses. While Yarning was predominantly used to engage with Aboriginal and Torres Strait Islander people, some Yarning circles included non-Aboriginal participants such as health providers.

#### Research team positioning

Generally, details regarding the research team positioning were not available in publications (see: Table 1 & supp. 1). Exemplar papers described the research team and their positioning and demonstrated reflexivity on how this influences all stages of research (see Table 2, 3.1). In these instances, authors described their ability to have “deeper” conversations with their participants, and the importance of established relationships in Aboriginal and Torres Strait Islander research. Some publications acknowledged that this contributed to providing a safe and favourable environment for participants.

**Table 1:** Included articles

**Author**	**Reporting Level**	**Primary Reference**	**Authorship** **Indigenous involvement**	**Indigenous senior authorship reported** **(1**^**st**^** or last)**	**Advisory**	**Location**	**Ethics**
**Canuto et al. (2019) **[40]	HIGH	Y	Y	Y	Y	One of four catchment areas of Yalata, Coober Pedy, Port Lincoln and metropolitan Adelaide	Aboriginal Health Council of South Australia’s Aboriginal Health Research Ethics Committee
**Munro et al. (2017) **[48]	LOW	Y	N	N	N	Service located in Western NSW	Aboriginal Health and Medical Research Council (AH&MRC)
**Meiklejohn et al. (2017) **[58]	MEDIUM	Y	Y	N	N	Brisbane, Australia	Human Research Ethics Committees of Northern Territory Department of Health, Menzies School of Health Research; Queensland Health, Darling Downs Hospital and Health Service; and QIMR Berghofer Medical Research Institute and participating Aboriginal community-controlled primary health care services
**Hamilton, Reibel et al. (2020) **[27]	MEDIUM	Y	Y	N	N	Banksia Hill Detention Centre (Banksia), youth detention facility in WA	Western Australian Aboriginal Health Ethics Committee
**Marriott et al. (2019) **[28]	HIGH	Y	Y	N	Y	Noongar Nation referred to as Noongar Boodjar (Noongar Country); located in the south of Western Australia	Ethics approval was sought from: WA Aboriginal Health Ethics Committee, and the Murdoch University, South Metropolitan Health Service and North Metropolitan Health Service and WA Health Country Health Service Ethics Committees
**Bryce et al. (2020) **[42]	HIGH	Y	Y	Y	N	Anangu Pitjantjatjara Yankunytjatjara lands (26 desert communities in the tristate region of Western Australia, South Australia, and the Northern Territory)	Ethics approval for this study was obtained from the Aboriginal Health Council of South Australia Incorporated
**Lin et al. (2017) **[43]	HIGH	Y	Y	N	Y	The project was con-ducted on Yamaji country	Ethics approval was granted through the Western Australian Aboriginal Health Ethics Committee
**Gonzalez et al. (2020) **[47]	MEDIUM	Y	Y	N	N	Kamilaroi community in the Tamworth New South Wales region	Hunter New England Local Health District (HNELHD) Human Research Ethics Committee and the Aboriginal Health and Medical Research Council (AH&MRC) Human Research Ethics Committee
**Hamilton, Maslen et al. (2020) **[68]	LOW	Y	Y	N	N	participants were from urban WA, five were from remote areas, and four from regional areas	Non-Aboriginal specific ethics
**Lin et al. (2013) **[55]	HIGH	Y	Y	N	Y	The research was undertaken in three towns in Western Australia: a regional town assigned the pseudonym of ‘Regiontown’ and two remote towns ‘Goldstone’ and ‘Desertedge’	Western Australian Aboriginal Health Ethics Committee and Curtin University Human Research Ethics Committee
**Kendall et al. (2019) **[59]	HIGH	Y	Y	Y	Y	A combination of minimum, medium, and maximum security prisons located in urban and regional locations in NSW were included	Aboriginal Health and Medical Research Council NSW
**Lin et al. (2012) **[56]	MEDIUM	Y	Y	N	Y	The research was undertaken in three towns in Western Australia: a regional town assigned the pseudonym of ‘Regiontown’ and two remote towns ‘Goldstone’ and ‘Desertedge’	Western Australian Aboriginal Health Ethics Committee and Curtin University Human Research Ethics Committee
**Lin et al. (2014) **[44]	MEDIUM	Y	Y	N	Y	The research was undertaken in three towns in Western Australia: a regional town assigned the pseudonym of ‘Regiontown’ and two remote towns ‘Goldstone’ and ‘Desertedge’	Ethical approval was sought and granted by the Western Australian Aboriginal Health Ethics Committee
**Kong et al. (2020) **[39]	HIGH	Y	Y	N	N	This study was conducted in the Greater Western Sydney region in NSW, Australia	Aboriginal Health & Medical Research Council
**Lyall et al. (2020) **[41]	LOW	Y	Y	N	Y	health service located in Inala—a southwestern suburb of Brisbane	Non-Aboriginal specific ethics
**Rix et al. (2014) **[62]	MEDIUM	N	Y	N	Y	A health district in rural New South Wales, Australia	The Aboriginal Health & Medical Research Council of New South Wales HREC
**Carlin et al. (2019) **[29]	MEDIUM	Y	Y	N	N	Pilbara—northern Western Australia	Western Australian Aboriginal Health Ethics Committee
**Schoen et al. (2010) **[63]	MEDIUM	N	Y	N	Y	A northern suburb of Perth, Moora, Tambellup and Marr-Mooditj Aboriginal Health Training College in Perth	Western Australia Aboriginal Health Information and Ethics Committee
**Butler et al. (2020) **[30]	HIGH	Y	Y	Y	N	five PHCCs across Queensland, New South Wales and Northern Territory	Aboriginal Health and Medical Research Council of New South Wales (AH&MRC) Ethics Committee
**Seear et al. (2019) **[49]	MEDIUM	Y	Y	N	N	Derby, a remote town in North West Australia	Western Australian Aboriginal Health Ethics Committee, Kimberley Aboriginal Health Planning Forum Research Subcommittee
**Gibson et al. (2018) **[46]	MEDIUM	Y	Y	N	N	A town and its surrounding area, on Wiradjuri country	“Two Human Research Ethics Committees (HREC) approved the research project. One of which specialises in Aboriginal health research”
**Cullen et al. (2020) **[31]	HIGH	Y	Y	Y	Y	Waminda South Coast Women’s Health and Welfare Aboriginal Corporation, located on the South Coast of NSW on Yuin Country; Katungul Aboriginal Corporation Regional Health and Community Services, located on the far South Coast of NSW on Yuin Country; Illawarra Aboriginal Medical Service on Dharawal Country; and Yerin Aboriginal Health Services, located on the NSW Central Coast on Darkinjung Country	NSW Aboriginal Health and Medical Research Committee
**Ryder et al. (2021) **[70]	MEDIUM	Y	Y	Y	N	South Australian, New South Wales and Queensland	Aboriginal Health and Medical Research Council, Aboriginal Health Research Ethics Committee
**Reibel et al. (2015) **[60]	HIGH	Y	Y	Y	y	WA	four Human Research Ethics Committees, including the WA Aboriginal Health Ethics Committee
**Bovill et al. (2019) **[65]	HIGH	Y	Y	Y	Y	Hunter New England area	AH&MRC Ethics Committee, Hunter New England Health Ethics Committee, University of Newcastle Ethics Committee
**Coombes et al. (2020) **[32]	MEDIUM	Y	Y	Y	N	South Australia, the Northern Territory, Queensland and New South Wales, Australia, from city, urban, remote and very remote areas	Non-Aboriginal specific ethics
**Coombes et al. (2018) **[33]	HIGH	Y	Y	Y	N	New South Wales (NSW)	Aboriginal Health and Medical Research Council (AH&MRC) Ethics Committee
**Bovill et al. (2019) **[65]	HIGH	Y	Y	Y	Y	NSW, SA and Qld	AH&MRC Ethics Committee
**Lukaszyk et al. (2017) **[45]	HIGH	Y	Y	N	N	Sydney, the Central Coast, Central West and Illawarra Shoalhaven, NSW	Aboriginal Health & Medical Research Council of NSW (AH&MRC)
**Durey et al. (2016) **[34]	MEDIUM	Y	Y	Y	Y	Perth—Armadale, Bentley, Fremantle, Rockingham-Kwinana and Peel (Mandurah)	Western Australian Aboriginal Health Ethics Committee
**Busija et al. (2018) **[50]	MEDIUM	N	Y	N	Y	Southern Downs Local Government Area (LGA), Queensland, Australia. Traditional Custodians of the area are the Githabul (Bundjalung nation) people and (on its Western edge) the Ngarabal people	Non-Aboriginal specific ethics
**Carlin et al. (2020) **[61]	HIGH	Y	Y	N	Y	Broome shire in the Kimberley region	Western Australian Aboriginal Health Ethics Committee
**Deacon-Crouch et al. (2016) **[51]	MEDIUM	Y	Y	N	Y	A Victorian regional Aboriginal Health Service	Non-Aboriginal specific ethics
**Helps & Barclay (2015) **[67]	MEDIUM	Y	Y	N	Y	A rural Aboriginal Community	Aboriginal Health and Medical Research Council ethics committee
**Chapman et al. (2014) **[35]	MEDIUM	Y	Y	N	N	“Victorian Emergency Department for the local ATSI community”	Non-Aboriginal specific ethics
**Meiklejohn et al. (2018) **[52]	MEDIUM	Y	Y	N	N	Queensland	Non-Aboriginal specific ethics
**Pilkington et al. (2017) **[53]	MEDIUM	Y	Y	N	Y	Metropolitan, rural and remote locations around WA	Western Australian Aboriginal Health Ethics Committee
**Rix et al. (2015) **[36]	LOW	Y	N	N	Y	Rural region of New South Wales, Australia—Bundjalung Nation	The Aboriginal Health and Medical Research Council of NSW
**Butten et al. (2019) **[57]	LOW	Y	Y	N	N	Caboolture—Northern Brisbane	Non-Aboriginal specific ethics
**Southcombe et al. (2015) [**69**]**	LOW	N	N	N	N	Urban, rural and remote regions in Australia	Non-Aboriginal specific ethics
**Henwood et al. (2017) **[71]	LOW	N	Y	N	N	Urban, regional and remote Australia	Non-Aboriginal specific ethics
**Belton et al. (2018) **[72]	LOW	Y	Y	N	N	Northern Territory	Non-Aboriginal specific ethics
**Meiklejohn et al. (2019) **[37]	LOW	N	N	N	N	Small regional community in Queensland	Non-Aboriginal specific ethics
**Murrup-Stewart et al. (2021) **[54]	LOW	Y	Y	Y	N	Narrm (Melbourne)	Non-Aboriginal specific ethics
**Peake et al. (2021) **[66]	LOW	Y	Y	N	N	Aboriginal communities in Northern New South Wales	Non-Aboriginal specific ethics
**Parmenter et al. (2019) **[38]	LOW	Y	N	N	N	Urban South-East and central Queensland	Non-Aboriginal specific ethics

**Table 2:** Descriptive quotes: how Yarning is being applied to qualitative health research

**Theme**	**Descriptor**	**Example**
1. Why Yarning?	1.1 Privileging Indigenous knowledges	The Indigenous voice in research has continually been suppressed and using an Indigenous data collection tool such as yarning is one vehicle through which the knowledges and values important to Indigenous participants can be prioritized in research. (Hamilton, Reibel et al. 2020 [27])
	1.2 Preferred method for Aboriginal community	Preferred research method for the older Aboriginal community. (Gibson et al. 2018 [46])
	1.3 Flexibility	Yarning is arguably more flexible than many conventional interviewing approaches, even though overlap and compatibility with some conventional methods is apparent. (Hamilton, Reibel et. al 2020 [27])
2. Reporting of Data Collection Processes	2.1 Rapport and comfortability	When women entered the room for the yarning circles, conversations began with a social yarn, establishing relationships and building trust between the women and the female researchers. (Bovill, Bar-Zeev et al. 2019 [65])
		Through social yarning, the intent is deeper, achieved through purposeful exchanges and trust building, in which the researcher shares information about themselves with the participant, and the level of information exchange is controlled by the participant. Throughout this process, the continuing responsibility of the researcher is to find shared ground through authentic interest in participant’s lives. This might be achieved through sharing information about culture and family, sports, hobbies, or interests. The research remains flexible to finding the shared ground, which requires some knowledge about and empathy toward the participant’s circumstances. (Hamilton, Riebel et al. 2020 [27])
		..typically begun with a ‘social yarn’ in which investigators established or reaffirmed an interpersonal connection, before moving on to a ‘research yarn’. (Lin, O’Sullivan et al. 2013 [55])
	2.2 Recording Equipment	To minimize potential harm to participants, a voice recording device was not used during yarning. Brief handwritten notes were taken during the interview, with salient points recorded in writing (verbatim) and doublechecked with participants for accuracy at the time of the interview. Immediately following the yarn, the researcher comprehensively documented details of the interview, including multiple reflective field notes. (Hamilton, Reibel et al. 2020 [27])
	2.3 Interview Guide	Participants were encouraged, with consistent prompts to ‘yarn’ about their experiences with renal service providers. (Rix, Barclay et al. 2014 [62])
		..minimal questioning or use of probes. (Bryce, Scales et al. 2020 [42])
	2.4 Seating arrangement	Most yarns were undertaken with the researcher and participant seated side by side with a respectful distance between and an intent to maintain a natural and nonthreatening engagement. At the same time, the proximity allowed the researcher to observe the participant’s body language, demeanors, and other nonverbal cues. (Hamilton, Reibel et al. 2020 [27])
3. Research Team Positioning	3. 1 Description of researchers involved	The remaining members of the research team are all Aboriginal men who work within Aboriginal and Torres Strait Islander health and have children of their own. The fact that the researchers were all Aboriginal or Torres Strait Islander fathers allowed for common ground with the participants and an ability to have far deeper and more frank conversations with the participants before, during and after the recorded yarning group sessions. At each of the yarning group sites, at least one member of the research team either had an established relationship with some or all of the participants which created favourable and safe interview conditions. (Canuto, Towers et al. 2019 [40])
	3.2 Researcher trained in Yarning	The research team have all undertaken training in yarning methods with Professor Dawn Bessarab, who is a Bard/Yjindjabandi woman and an expert in Indigenous research, qualitative methodologies and yarning methods (Cullen, Mackean et al. 2020 [31])

Training and experience in both qualitative and Indigenous methods were often not reported. Eleven of the publications that reported Aboriginal and/or Torres Strait Islander involvement in data collection also outlined the experience, qualifications and expertise of the interviewer/s (see Table 2, 3.2) [33, 39, 40, 42, 45, 50, 51, 60, 64, 65, 72]. Six of the publications specifically stated that the interviewers had relevant training in conducting qualitative interviews [31, 33, 45, 50, 51, 72]. One publication [31] specifically detailed that the research team had been trained in Yarning methods with Professor Dawn Bessarab, who validated the method. This was a more comprehensive example of the way training was reported compared to the way training was reported in other publications.

### The role of Aboriginal and Torres Strait Islander people in the Yarning process

We sought to detail the role of Aboriginal and Torres Strait Islander people in each stage the research process in publications that use Yarning method. In particular, we examine the role of Aboriginal and Torres Strait Islander people in leading the research, collecting data, analysis, and acting as an advisory to the research.

#### Aboriginal & Torres Strait Islander led research

Eleven of the forty-seven publications reported Aboriginal and Torres Strait Islander people as having led the research [28, 30, 32–34, 40, 42, 54, 64, 65, 70]. Those that were led by Aboriginal and Torres Strait Islander academics occasionally offered details on the authors and their roles. This was detailed using the authors initials, followed by their Aboriginal status, positioning, and role in the project. However, these details were scarce and difficult to immediately identify within publications. More comprehensive papers reflected on how these factors created an approach that helped centre Aboriginal voices in the research process (see Table 3, 1.1).

**Table 3:** Descriptive quotes: The role of Aboriginal and Torres Strait Islander people in the Yarning Process

1. Aboriginal & Torres Strait Islander led research	1.1 Details on authors and their roles	“The Screening Matters study was conceptualised, led, and conducted by Indigenous Australian women: LJW, TB, GG, BM and two Aboriginal community research officers. It privileged the voices of Indigenous Australian women–the participants. Finally, the study aimed to understand the individual, community, and structural influences on Indigenous Australian women’s participation in cervical screening. Together, this approach ensured that Indigenous Australian women’s perspectives on cervical screening were centred in the research.” (Butler, Anderson et al. 2020 [30])
2. Research Team Positioning	2. 1 Description of researchers involved	The remaining members of the research team are all Aboriginal men who work within Aboriginal and Torres Strait Islander health and have children of their own. The fact that the researchers were all Aboriginal or Torres Strait Islander fathers allowed for common ground with the participants and an ability to have far deeper and more frank conversations with the participants before, during and after the recorded yarning group sessions. At each of the yarning group sites, at least one member of the research team either had an established relationship with some or all of the participants which created favourable and safe interview conditions. (Canuto, Towers et al. 2019 [40])
	2.2 Researcher trained in Yarning	The research team have all undertaken training in yarning methods with Professor Dawn Bessarab, who is a Bard/Yjindjabandi woman and an expert in Indigenous research, qualitative methodologies and yarning methods. (Cullen, Mackean et al. 2020 [31])
3. Aboriginal Involvement in data collection	3. 1 Detailed description of Aboriginal researcher	The research was led by a Torres Strait Islander man (KC), who is an experienced qualitative researcher. The yarning groups were co-facilitated by KC and an Aboriginal male health worker and health service team leader (KT). KT led the yarning group discussions using a semi-structured yarning guide. (Canuto, Towers et al. 2019 [40])
		Qualitative data were collected through use of yarning methodology between August 2015 and January 2016 by a female Aboriginal Researcher (MB) with experience and qualifications in social and community services. (Bovill, Gruppetta et al. 2018 [64])
	3.2 Limited description Aboriginal researcher	The qualitative researcher was an Aboriginal woman, making yarning culturally safe and aligned with the cultural values of Aboriginal people. (Hamilton, Maslen et al. 2020 [68])
	3.3 Non-Aboriginal researcher with limited discussion	Interviews were conducted in person at OH by a non-Aboriginal female researcher. (Munro, Allan et al. 2017 [48])
		The interviewer was a female non-Indigenous graduate research student living in Derby. (Seear, Lelievre et al. 2019 [49])
	3.4 Reflexive discussion for no Aboriginal involvement	Despite attempts and available funding, we were unable to secure a Pilbara Aboriginal co-researcher to work alongside us during the design, interviewing and analysis of this study. This was, in part, due to the existing workloads and other responsibilities of Aboriginal people in the Pilbara who were interested in and supportive of the project. In the absence of an Aboriginal co-researcher, our relationship with Aboriginal staff at recruitment sites and the Pilbara Aboriginal Health Planning Forum (PAHPF) was crucial in obtaining feedback on the research design, approach and analysis, and ensuring that the study was undertaken in a culturally safe way. (Carlin, Atkinson et al. 2019 [29])
		Both researchers were present for the group sessions and for 3 of the interviews. The other 9 interviews were conducted by the non-Indigenous researcher independently. No difference in data, in terms of collection or results, was perceived by the non-Indigenous researcher when conducting the interviews. (Butten, Johnson et al. 2019 [57])
	3.5 Yarning as a way of addressing not having Aboriginal involvement	Although in this study, non-Indigenous researchers convened ‘focus group’ discussions (the term ‘focus group’ will be used for consistency), they became yarning circles where the Aboriginal health staff exchanged knowledge about their own perspectives and personal views of Aboriginal women’s experiences through shared stories in an Aboriginal way. This exchange, using yarning, changed the dynamic of the focus group so that the non-Indigenous researchers would learn from the Aboriginal health staff. (Kong, Sousa et al. 2020 [39])
		This approach facilitated a dynamic exchange where Aboriginal knowledge could be taught and shared by the Aboriginal staff to the non-Indigenous researchers, building trust and reciprocity. (Kong, Sousa et al. 2020 [39])
	3.6 Aboriginal guidance to account for not having Aboriginal involvement	Access to the sites was organised by the Indigenous representative, who was culturally aware and sensitive to the needs of the participants, and he accompanied the researchers and assisted in the interview and yarning circle procedures. In this regard, the researchers were cognisant of the history of ‘exploitative and harmful research practices’, in the context of non-Indigenous researchers ‘working with Indigenous peoples and communities’. (Henwood, Shaw et al. 2017 [71])
		The possibilities for power imbalances were acknowledged during recruitment and participant interviews and all attempts were made to identify ways to minimise this occurring. Ongoing guidance was sought and appreciated from the AH&MRC ethics committees, Aboriginal advisors and other researchers experienced in Indigenous research. (Helps and Barclay 2015 [67])
4. Aboriginal & Torres Strait Islander Involvement in Analysis	4.1 Collaboration between Aboriginal and non-Indigenous Investigators	Aboriginal investigators explained the differences using their cultural knowledge, which led the non-Aboriginal investigators to read the women’s data differently, using different ‘lenses’. (Marriott, Reibel et al. 2019 [28])
		Initial summaries of the data were reviewed by members of the interprofessional research team (physiotherapy, Aboriginal health, public health medicine and anthropology), and Aboriginal coinvestigators to include perspectives, themes and issues that might not otherwise have been considered. (Lin, O'Sullivan et al. 2013 [55])
		Coding was undertaken concurrently throughout data collection by JM who conducted the yarns to assist early coding and inform ongoing data collection. In addition, an analysis meeting was held with four members of the research team (JM, BM, BA, and CMB), two of whom identify as Indigenous, to refine categories and patterns across the stories as well as to seek agreement on identified categories. (Kong, Sousa et al. 2020 [39])
	4.2 Opportunity for feedback from participants	After agreement between AK, MSS and FT, AK convened with the AHWs and FPWs separately to yarn about the themes. This allowed for participants to check, engage and further contribute to the interpretation of the data, and ensure rigor. (Kong, Sousa et al. 2020 [39])
	4.3 Indigenous approach to Analysis	To guide this process we drew on the research practice described in Dadirri—an Indigenist research approach that calls for researchers’ deep listening for what is being communicated, along with what is not shared. A commitment to our critical reflexivity for how we listened to and analyzed participants’ stories was pivotal to this process, as was mindfulness of the local, national, and historical contexts within which participants’ stories were being shared. (Lyall, Guy et al. 2020 [41])
		Although critiques of Yarning point to difficulty in establishing rigor due to the inherent “messiness” of gathered data, these limitations are based on perspectives from Eurocentric epistemological priorities which can be offset by establishing Indigenous epistemological foundations and engaging an appropriate cultural lens to analysis. (Murrup-Stewart, Whyman et al. 2021 [54])
5. Advisory	5. 1Detailed description of advisory group and their role	A study reference group (SRG) made up of representatives from supporting organisations and services was established. The SRG members are Aboriginal and Torres Strait Islander people (men and women), including Aboriginal and Torres Strait Islander male community members and one non-Indigenous male. The Aboriginal and Torres Strait Islander men of the SRG guided the research team throughout the research process; their guidance ensured the research was conducted appropriately. (Canuto, Towers et al. 2019 [40])
	5.2 Limited description of advisory group and their role	An Aboriginal Reference Group was established to provide guidance, cultural advice and input into the information and processes of the research. (Schoen, Balchin et al. 2010 [63])

#### Aboriginal & Torres Strait Islander involvement in data collection

Aboriginal and Torres Strait Islander researchers were reported as responsible for data collection and interviews in only half of the publications [71]. Some publications provided less detail on data collection involvement than others, and simply noted that the researcher was Aboriginal and therefore culturally safe (see Table 3, 3.2).

Seven publications reported that interviews were conducted by both Aboriginal and/or Torres Strait Islander and non-Aboriginal and/or Torres Strait Islander researchers, [28, 31, 39, 56, 57, 59, 63] often citing that one was there to assist the other. One publication identified that there was *“no difference in data, in terms of collection or results, was perceived by the non-Indigenous researcher when conducting the interviews.”* [57].

Six publications reported that there was no Aboriginal and/or Torres Strait Islander involvement in data collection and the conducting of Yarning circles [29, 38, 48, 49, 67]. Some publications did not expand on this, and simply stated that the data collection was conducted by a non-Aboriginal researcher without further discussion (see Table 3, 3.3). Others were more reflexive when addressing not having had an Aboriginal and Torres Strait Islander person collect the data. One publication [29] stated that they attempted to have an Aboriginal and Torres Strait Islander researcher involved, however, due to existing responsibilities of Aboriginal people in the area, they were unsuccessful (see Table 3, 3.4). This publication then addressed the absence of Aboriginal involvement by detailing the critical part that relationships with the staff at Aboriginal Health Services were to different stages of the project. Another paper stated that the lack of Aboriginal involvement *“may have impacted on the richness of interview data”* [48].

Whilst rare, some papers suggested Yarning methods as an effective way to counter the impact of not having an Aboriginal or Torres Strait Islander person collect the data (see Table 3, 3.5).

Others accounted for the lack of Aboriginal and/or Torres Strait Islander involvement in data collection through using Aboriginal guidance over the project, stating ongoing guidance was sought throughout various stages of the project (see Table 3, 3.6).

Overall, the absence of Aboriginal and Torres Strait Islander involvement in the collection of data was frequently not addressed by publications. Eighteen publications did not report whether or not there were Aboriginal and/or Torres Strait Islander involvement in data collection [28, 31, 32, 34, 36, 37, 41, 46, 47, 52–54, 58, 62, 66, 69, 70, 72]. For the most part, these publications did not identify who was responsible for conducting the Yarning circles.

#### Aboriginal & Torres Strait Islander involvement in analysis

Twenty-four of the 46 publications reported Aboriginal and/or Torres Strait Islander involvement in analysis [28–34, 39, 40, 43, 45, 47, 49, 50, 52, 55, 56, 58–62, 64, 65]. Predominately it was simply stated that there were Aboriginal and/or Torres Strait Islander researchers or advisory groups involved in the process, without further elaboration of exactly what the involvement entailed. One paper suggested that Aboriginal and/or Torres Strait Islander investigators helped non-Indigenous researchers to *“increase their cultural understandings and read the data differently.*” [28]. Other publications reported a collaborative analysis revision by experts and Aboriginal and/or Torres Strait Islander coinvestigators (see Table 3, 4.1). This was deemed efficient in the inclusivity of perspectives that may have not been considered.

Many publications did not report whether or not they had Aboriginal and/or Torres Strait Islander involvement in data analysis. Some of these included publications that used the researchers initials to demonstrate involvement in analysis but did not specify whether they were Aboriginal and/or Torres Strait Islander. Not having Aboriginal and/or Torres Strait Islander involvement in data collection was typically not reflexively addressed.

One publication did report not having Aboriginal and/or Torres Strait Islander involvement in data collection and identified potential issues with this throughout their publication with reflections such as *“The challenge for a non-Aboriginal researcher exploring issues within the Aboriginal community is to avoid repeating mistakes of the past.”* [67] Other publications noted (by those who acknowledge it) that Aboriginal and/or Torres Strait Islander advisory, person or group was used to overlook data analysis.

A limited number of publications indicated that participants were offered the opportunity to provide feedback on the findings (see Table 3, 4.2). It was often reported by simply stating that data was returned to participants for feedback. Some publications elaborated on this, with one suggesting that yarning with participants about the results allowed for them to review and engage with the interpretation of data. Some publications noted that transcripts were not returned to participants, nor were the data validated by participants without further comment.

The few publications that reported using an ‘Indigenous approach’ to analysis were slightly more comprehensive than those that reported grounded theory approach (see Table 3, 4.3). Overall, details on analysis were often lacking, and were the least comprehensive component of the methodology sections.

#### “Aboriginal Advisory”

Only twenty-nine of the 46 included publications reported having an Aboriginal and/or Torres Strait Islander advisory, person or group throughout the course of their study [27–29, 31, 33, 34, 36, 37, 39–43, 45–47, 51, 53–56, 59, 60, 62, 63, 65–68]. Advisory groups were used to oversee the research, analyse data, develop protocols, guide research conduct, identify potential services and recruitment, and develop interview guides. Publications that offered a more comprehensive description of the role of the advisory group gave details on who was involved, such as Elders and community members, and how their guidance was utilised in each stage of the research process (see Table 3, 5.1). Often, these publications noted that the reference groups held the study team accountable for conducting appropriate and respectful research.

Most publications reported an Aboriginal reference or guiding group, with limited details on who was involved, or what exactly their role entailed (see Table 3, 5.2). Many of the publications did not mention Aboriginal and/or Torres Strait Islander Advisory until the acknowledgment section of the manuscript. The process of Aboriginal and/or Torres Strait Islander advisory varied across publications, with limited consistency in the level of reporting and who was involved.

## Discussion

This is the first review to critically analyse the use of Yarning method in Aboriginal and Torres Strait Islander health research. Through this, we make recommendations on how systems, including the Academy and other mechanisms such as journals, can better incorporate Aboriginal and Torres Strait Islander ways of knowing, being and doing into systems and processes, to ultimately uphold research integrity.

Although there is a strong and growing evidence-base for Indigenous quantitative methods that have been used by Indigenous scholars with ongoing room for improvement in everyday practice, [73] researchers using Indigenous methods in health research frequently report using qualitative methods [13]. Qualitative methods are said to privilege Indigenous voices [12] and remove power imbalances [13, 18]. Yarning method is the most commonly reported Indigenous method applied to Aboriginal and Torres Strait Islander qualitative health research. Despite this, our analysis shows that details regarding how Yarning methods were applied, and the intricacies of Aboriginal and Torres Strait Islander involvement (such as stages, level or type of involvement), were significantly under reported. While part of this may be attributed to limitations in researcher reflexivity, the level of detail required to situate authors positionality, relationality as well as thoroughly describe research processes are not always achievable within the existing parameters of journal and reporting guidelines. We offer our recommendations and improvement opportunities for both researchers, and academic institutions to ensure reporting in publications reflects the need for ethical and reciprocal research with Aboriginal and Torres Strait Islander people, as per the NHMRC Values and Ethics Guideline, [14] the AIATSIS Code of Ethics [15] and the AHMRC Key Principles [16].

### The right reasons? Why are researchers using Yarning methods?

**Fig. 3:**
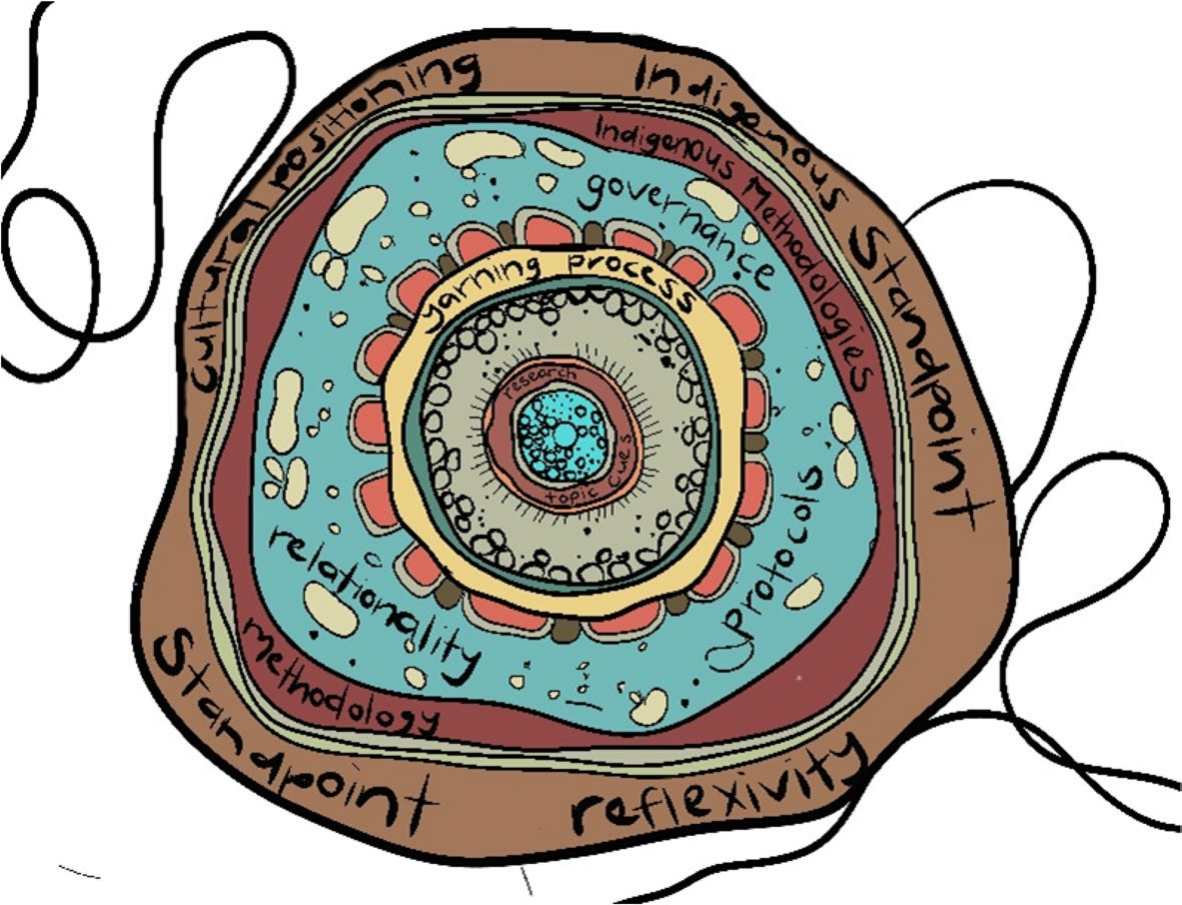
Visual representation of the Yarning Process in line with decolonising research practice as depicted by Michelle Kennedy

In the reviewed publications, Yarning was frequently cited as a way of decolonising research practice. It was considered culturally safe, offer two-way knowledge sharing, built on cultural protocols, and allow participant led research while attempting to better balance and privilege Indigenous voices. Numerous research has validated Yarning as a recommended method to privilege Indigenous ontologies [9, 17, 18]. However, it is not simply enough to report employing an Indigenous method such as Yarning and assume that it is adequate. Yarning is grounded in cultural positioning [17] and relationality [11]. Therefore, the application of Yarning will vary based on the context and the researcher (including their social and cultural positioning, and considerations of power and control) and the Aboriginal and Torres Strait Islander community involved. Similarly, Yarning is not simply the means to collect the data. Decolonising research must address the research process as a whole and centre Indigenous worldviews, values and principles [1, 74]. This is depicted in Fig. 3.

### Reporting positioning, reflexivity and relationality is essential for ethical research

Irrespective of employing a decolonising approach, researcher positioning is well understood as a necessary component to conducting reflexive, ethical and quality research in all qualitative research practice. Researchers are embedded within the research process, and are therefore required to constantly consider their worldview and positionality [75]. As Kiekelame and Swartz (2019) conclude *“the importance of reflexivity and self-reflexivity as a transformative approach in a decolonising process cannot be over emphasised”* [76]. Despite researcher positioning and reflexive practices being at the core of qualitative research *and* the importance of Aboriginal and Torres Strait Islander led research, we found limited information reported on the research team’s context, including social and cultural positioning and relationality which is a central “presupposition of an Indigenous social research paradigm”; [11] our belonging to Mob and Country, the connection to the living earth must be recognised and strategically mobilised by Indigenous peoples in developing an Indigenous research agenda.

While some publications articulated social and cultural positioning and relationality of researchers and decolonising research practice, most were silent on these aspects. This silence and subsequent invisibility, often leads to reinscribe racial dominance in theorising, analysing and undertaking research in practice; embedded through the ongoing experience of colonisation and the ingrained nature of coloniality across Australia.

Non-Indigenous authors did not always detail reflective practice or identify their social and cultural positioning. This is in opposition to decolonising research practice which seeks to address Euro-Western dominant paradigms [1, 77]. Describing Aboriginal and Torres Strait Islander involvement, researcher reflexivity and positionality, and relationalities are crucial in research, including *both* qualitative processes *and* decolonising approaches. This is even more critical when Indigenous methodologies and methods are reportedly being applied by non-Indigenous peoples. Publications that reported using an “Indigenous lens” rarely articulated how this was applied, and how it informed the publication. While this can partially be attributed to structural publishing barriers, such as word count limitations, it is essential for researchers to describe how an Indigenous lens was applied as well as their role and how their perspectives inform the research process. It is not enough to note the application of an “Indigenous lens” but also how they applied this lens, particularly from a non-Indigenous standpoint. Reflecting on how their own practices, world views and experiences impacted and influenced the research outcomes and effectively and accurately represented the voices of participants in the research. Accountability in qualitative research requires the application and description of reflexive research practices in relation to the researcher and the researched. It is not possible for a researcher to completely omit researcher bias. It is therefore imperative to outline the reflexive processes, and how Indigenous peoples informed the research in a comprehensive manner in favour of upholding culturally safe, ethical and best practice qualitative research.

### Aboriginal and Torres Strait Islander people should be involved in all stages of the research process

Despite ongoing calls for, and emphasis on the importance of having research to be led by Aboriginal and Torres Strait Islander people, only a quarter of publications self-reported Aboriginal or Torres Strait Islander people as lead researcher. The remaining papers were silent in reporting Aboriginal and/or Torres Strait Islander researcher(s) involvement or were led by non-Indigenous researchers. The transformative nature of Indigenous-led research is well established, [1] as such we urge all researchers to report leadership of the research in the publication.

Just over half the papers reported Aboriginal and/or Torres Strait Islander people’s involvement in data collection, with limited information provided on qualifications/training or the relationship and cultural expertise to the participants or community engaged in the research. Acknowledging the centrality of following cultural protocols and practices when conducting Yarning method, details on data collection must report this detail when applying the method.

Aboriginal and/or Torres Strait Islander involvement in analysis was seldom detailed, more frequently papers reported advisory groups, increasing cultural understanding, with and oversight as their primary role. Researchers and coders play a pivotal role in the process of thematic analysis [78]. Detailing Aboriginal and Torres Strait Islander involvement in all levels of the research conduct including the analysis is paramount to accurately representing the voices of those being researched and supporting ethical and cultural safe research. Decolonising research practice must privilege Indigenous knowledges and uphold self-determination and sovereignty which is not excluded from the analysis and reporting process. In line with recent research into Yarning application, [19] we recognise the need for refinement in the analysis process, and the importance of producing details of method and methodologies used.

As stated by Atkinson et al. [19] *“the more relational the Yarn, the greater the thickness of data, and an Indigenous Standpoint is likely to generate more relationality through shared implicit and explicit understanding for the Yarn”.* Our research demonstrates that the relationality of Yarns is not consistently carried beyond the stages of data collection and into analysis, or at least, is not reported on.

We found that at times, Aboriginal research assistants were used to collect the data, but were not involved in the interpretation of the Yarns. To produce ethical, quality research, Aboriginal people should be involved in all stages of the research from conceptualisation to dissemination, including the analysis and interpretation. Understanding and conceptualising data collected from Yarning should involve Indigenous ontologies and standpoints to ensure participants stories are correctly and appropriately reported in the research results.

### The role and details of Aboriginal reference/Advisory groups need to be reported

The formation of a community advisory or Aboriginal reference group (CAG/ARG) is important in ensuring governance and efficacy in the research process, as well as upholding Indigenous knowledges, sovereignty, and self-determination. The Aboriginal Health & Medical Research Council ethical guidelines state that Aboriginal and Torres Strait Islander ‘Advisory’ or reference groups *must be representative of the group being studied and have knowledge or experience of the research matter* and *must be engaged throughout the life cycle of the project not just at the development or consultation stage* [16].

While the majority of publications reported having a CAG/ARG that provided oversight to the research process, we found that publications reported limited details on who was involved, and their specific role, particularly regarding analysis, reporting and validation of results. This detail is critical to understanding how the CAG/ARG is both representative of the group being studied and how their Indigenous knowledge, self-determination and sovereignty were upheld in the research process. While some publications offered detailed explanations of who was in the advisory group and what their role was through the research process, others simply stated that the research was overseen or guided by an Aboriginal advisory group. Offering details on the CAG/ARG is crucial, particularly when non-Indigenous researchers are engaging with Indigenous methods, such as Yarning. We question: Who validates that the methods are applied correctly? Is this left to the researcher to self-validate? And where is the research team’s accountability to the Aboriginal and/or Torres Strait Islander community being studied?

Aboriginal and Torres Strait Islander “Advisory” or reference group are critical to ethical research practice, and must not be used to rubber stamp the research process. Research that aims to improve the lives of Aboriginal and Torres Strait Islander peoples must foreground Indigenous knowledges, sovereignty, and self-determination through relationality. Watego asserts *“The transdisciplinarity required to effect change requires more than a bringing together of different methodologies—it demands attention to different ways of knowing and being in a relational, rather than hierarchical, manner, recognising the limitations of different knowledge systems as well as their strengths, so that the most appropriate conceptual tools are brought to bear in addressing the grand challenges we face both now and into the future” *[79].

### Academic institutions and journals require structural change to account for reporting

As detailed throughout the results and discussion section of this review, authors frequently omitted important details regarding standpoint, positioning, reflexivity, level of Aboriginal and Torres Strait Islander involvement, and explanations of methods. It is reasonable to assume that silence in some of these areas are due to barriers in publishing. Academic journals should cater to the need to report reflexivity and positionality, particularly in relation to Indigenous research, including Aboriginal and Torres Strait Islander research. Many journals have restrictive word counts and journal structures and essential reporting requirements, which create barriers to effectively reporting adequate details that demonstrate best practice, ethical and equitable research. Academic journals and existing structures should require accurate reporting to produce community relevant, scientific excellence in quality and valid qualitative inquiry, that considers and contextualises findings to the local context. Additionally, it is important to move beyond reporting qualitative rigour as simply just a check box exercise. The Qualitative Health Research (QHR) journal recently released an editorial detailing why their review process does not use checklists:

*These lists ignore the value of the product of the research: They do not address the originality, the substance, the contribution, and the potential results to the actual topic—which is after all the purpose of the project itself.* [80]

The editorial explains that checklist reviews can undermine the value of qualitative inquiry [80]. This authorship team suggested that beyond checklists, journals acknowledge Indigenous knowledge systems and seek contribution of Indigenous peer reviewers on the reporting of Indigenous methodologies and methods to uphold the appropriate reporting requirements [81].

### Strengths and limitations

This paper reports a review of publications reporting the use of Yarning method in Aboriginal and Torres Strait Islander health research. Our review was led by an Aboriginal research team including the author of the Yarning as a legitimate research method publication [17]. Our review provides a critical analysis of Yarning method as applied to qualitative health research and provides guidance to researchers on the future use, and reporting of Yarning method. Whilst Yarning is a culturally safe method that is preferred by Aboriginal and Torres Strait Islander people, non-Indigenous researchers need to consider the significance of relationality, sovereignty, and integrity of the research in the doing through the inclusion of Indigenous leadership at every stage.

Authors note that the Yarning method is applied to other disciplines of research and as such this paper offers limitations to understanding it’s broader application. Some publications in this review also included the use of other Indigenous and/or decolonising methods which were not analysed and out of scope in this review. Further analysis on additional Indigenist methods would be insightful.

## Conclusion

Aboriginal and Torres Strait Islander people should be at the forefront of research about them. Coloniality has embedded systemic racism in our societal structures, privileging non-Indigenous peoples and disadvantaging Indigenous peoples. Coloniality perpetuates ideas about Indigeneity which are then formed and validated through social, cultural, and political structures, practices, and beliefs. They play out in our languages, knowledges, academic discourse, personal and social interactions and popular cultures, and other domains that assign and negotiate meanings and values [82]. Universities and research are not omitted from coloniality, which too, continue to systematically privilege non-Indigenous knowledge systems, methodologies and methods. Despite cutting edge research by Aboriginal and Torres Strait Islander people since time immemorial, the exclusion of Indigenous knowledges, ways of knowing being and doing has a lasting impact that extends to peer review publications processes and policy development. Euro-Western academic hierarchies, “gold standard” reporting do not necessarily allow for, or consider, Indigenous ways of knowing or uphold Indigenous sovereignty and self-determination in the research process. Although Yarning is recognised as a legitimate research method to decolonising research practice, this method must not be used lightly to justify safety and security in research with Aboriginal and Torres Strait Islander people. It must be applied rigorously and reported accurately, describing how the different types of yarning were applied in research, the involvement of Aboriginal and Torres Strait Islander peoples at all levels of the research, and the outcomes. We found that researcher reflexivity and positioning were significantly under detailed as was Aboriginal and Torres Strait Islander ownership, stewardship, custodianship and analysis of data collected in our reviewed publications. Researchers, particularly non-Indigenous led research teams, must only report using an Indigenous method if they are willing to report adequate detail on its application and comprehensive detail on how Aboriginal and Torres Strait Islander peoples were involved in all levels of the research. Journals and other establishments should review their process to allow for these details to be documented in research publications without penalty and acknowledge the critical role of Indigenous Editors and peer reviewers. Only through this, can we uphold respectful, reciprocal, ethical, and responsible research practice.

## Supplementary Information

**Additional file 1.** Level of reporting table and scoring system.

## Data Availability

All data generated or analysed during this study are included in this published article [and its supplementary information files].

## Abbreviation

CAG/ARG: Community advisory or Aboriginal reference group
