# Supplementary material for: Decolonising qualitative research with respectful, reciprocal, and responsible research practice: a narrative review of the application of Yarning method in qualitative Aboriginal and Torres Strait Islander health research
Source: Int J Equity Health. 2022 Sep 13;21:134. doi: 10.1186/s12939-022-01738-w (PMC9472448; doi:10.1186/s12939-022-01738-w)
Supplement: Supplementary file 1 — Additional file 1. Level of reporting table and scoring system. [file 12939_2022_1738_MOESM1_ESM.docx]

| Author, Year | Reference | Development | Data Collection | Experience | Analysis | Aboriginal Ethics | Score | Rating |
| --- | --- | --- | --- | --- | --- | --- | --- | --- |
| Canuto et al. (2019) | X | X | X | X | X | X | 6 | HIGH |
| Munro et al. (2017) | X |  |  |  |  | X | 2 | LOW |
| Meiklejohn et al. (2017) | X |  |  |  | X | X | 3 | MEDIUM |
| Hamilton, Reibel et al. (2020) | X |  | X |  |  | X | 3 | MEDIUM |
| Marriott et al. (2019) | X | X | X |  | X | X | 5 | HIGH |
| Bryce et al. (2020) | X | X | X | X |  | X | 5 | HIGH |
| Lin et al. (2017) | X | X | X |  | X | X | 5 | HIGH |
| Gonzalez et al. (2020) | X | X |  |  | X | X | 4 | MEDIUM |
| Hamilton, Maslen et al. (2020) | X |  | X |  |  |  | 2 | LOW |
| Lin et al. (2013) | X | X | X |  | X | X | 5 | HIGH |
| Kendall et al. (2019) | X | X | X |  | X | X | 5 | HIGH |
| Lin et al. (2012) | X | X | X |  |  | X | 4 | MEDIUM |
| Lin et al. (2014) | X |  | X |  | X | X | 4 | MEDIUM |
| Kong et al. (2020) | X |  | X | X | X | X | 5 | HIGH |
| Lyall et al. (2020) | X | X |  |  |  |  | 2 | LOW |
| Rix et al. (2014) | X | X |  |  | X | X | 4 | MEDIUM |
| Carlin et al. (2019) | X | X |  |  | X | X | 4 | MEDIUM |
| Schoen et al. (2010) |  | X | X |  |  | X | 3 | MEDIUM |
| Butler et al. (2020) | X | X | X |  | X | X | 5 | HIGH |
| Seear et al. (2019) | X |  |  |  | X | X | 3 | MEDIUM |
| Gibson et al. (2018) | X | X |  |  |  | X | 3 | MEDIUM |
| Cullen et al. (2020) | X | X | X | X | X | X | 6 | HIGH |
| Ryder et al. (2021) | X | X | X |  |  | X | 4 | MEDIUM |
| Reibel et al. (2015) | X | X | X | X | X | X | 6 | HIGH |
| Bovill et al. (2019) | X | X | X | X | X | X | 6 | HIGH |
| Coombes et al. (2020) | X | X |  |  | X |  | 3 | MEDIUM |
| Coombes et al. (2018) | X | X | X | X | X | X | 6 | HIGH |
| Bovill et al. (2019) | X | X | X | X | X | X | 6 | HIGH |
| Lukaszyk et al. (2017) | X | X | X | X | X | X | 6 | HIGH |
| Durey et al. (2016) | X | X |  |  |  | X | 3 | MEDIUM |
| Busija et al. (2018) |  | X | X | X | X |  | 4 | MEDIUM |
| Carlin et al. (2020) | X | X | X |  | X | X | 5 | HIGH |
| Deacon-Crouch et al. (2016) | X | X | X | X |  |  | 4 | MEDIUM |
| Helps & Barclay (2015) | X | X |  |  |  | X | 3 | MEDIUM |
| Chapman et al. (2014) | X | X | X |  |  |  | 3 | MEDIUM |
| Meiklejohn et al. (2018) | X | X |  |  | X |  | 3 | MEDIUM |
| Pilkington et al. (2017) | X | X |  |  |  | X | 3 | MEDIUM |
| Rix et al. (2015) | X |  |  |  |  | X | 2 | LOW |
| Butten et al. (2019) | X |  | X |  |  |  | 2 | LOW |
| Southcombe et al. (2015) |  |  |  |  |  |  | 0 | LOW |
| Henwood et al. (2017) |  | X |  |  |  |  | 1 | LOW |
| Belton et al. (2018) | X |  | X |  |  |  | 2 | LOW |
| Meiklejohn et al. (2019) | X |  |  |  |  |  | 1 | LOW |
| Murrup-Stewart et al. (2021) | X | X |  |  |  |  | 2 | LOW |
| Peake et al. (2021) | X | X |  |  |  |  | 2 | LOW |
| Parmenter et al. (2019) | X |  |  |  |  |  | 1 | LOW |
| Total | 42 | 33 | 26 | 11 | 23 | 21 |  |  |

**Supplementary File 1. Level of reporting table and scoring system**

0-2= low 3-4= medium 5-6 = high
